# The Role of Endothelium in Physiological and Pathological States: New Data

**DOI:** 10.1155/2018/1098039

**Published:** 2018-11-18

**Authors:** Agata Stanek, Bahar Fazeli, Stanisław Bartuś, Edyta Sutkowska

**Affiliations:** ^1^School of Medicine with the Division of Dentistry in Zabrze, Department of Internal Medicine, Angiology and Physical Medicine, Medical University of Silesia, Batorego St. 15, 41-902 Bytom, Poland; ^2^Immunology Research Center, Inflammation and Inflammatory Diseases Division, School of Medicine, Mashhad University of Medical Sciences, Mashhad, Iran; ^3^II Department of Cardiology, Jagiellonian University, Kraków, Poland; ^4^Department and Division of Medical Rehabilitation, Wrocław Medical University, Borowska 213, 50-556 Wrocław, Poland

Endothelium is the endocrine organ essential for maintenance of homeostasis in the entire body. Healthy endothelial cells regulate antioxidants, have anti-inflammatory and anticoagulant action, and, thus, control vascular relaxation and contraction, thrombogenesis, fibrinolysis, and platelet activation and inhibition. Maintaining the functional integrity of this complex organ is, therefore, critical for preserving blood flow and preventing thrombosis.

Endothelium dysfunction (ED) may result in the loss of important homeostatic functions, which, in turn, leads to various pathologies. ED has been observed in relation to ageing as well as in major lifestyle-related diseases, suggesting that endothelium dysfunction can serve as a means of identifying people for the purpose of preventing and treating various diseases. For example, ED is the initial stage in the pathogenesis of peripheral artery disease, cardiovascular diseases, chronic venous disease, chronic kidney failure, cancer, infectious diseases, and obesity.

This special issue comprises seven original research articles and two reviews, summarised in brief below, that delve into the important research being conducted on endothelial function.

The loss of endothelial integrity and function is a critical component of the onset of the adverse changes that result in cardiovascular disease. Injury of the endothelium is a consequence of haemodynamic (e.g., wall shear stress), chemical (e.g., low-density lipoprotein (LDL) cholesterol or glucose), or biologic factors (e.g., immune complexes). It is a known fact that mechanical factors, such as shear stress, are involved in the insult to the endothelial cells, but the level of this fluid drag force, which could damage the cells as well as the way they work, is still being studied. In the paper “Computing of Low Shear Stress-Driven Endothelial Gene Network Involved in Early Stages of Atherosclerotic Process”, F. Vozzi et al. study the impact of different shear stress levels on endothelial gene expression in vitro using a laminar flow bioreactor. The authors show that different shear stress levels have a significant influence on the activity of the endothelial cells and their metabolic profile. They also demonstrate that lower shear stress promoted the proliferation properties of the examined cells by flow-induced gene expression modulation of some endothelial genes (upregulation for the genes particularly linked to the inflammation and apoptosis versus downregulation for the contrary acting genes).

Likewise, loss of endothelial integrity permits lipid infiltration, which forms the soft lipid of the atheromatous plaque. Many factors influence the development of atheromatous plague, many of which (so-called “modifiable risk factors”) can be successfully treated. Amongst the applicable therapies are antiplatelet therapy and statin therapy. Both can result in very simple improvements, such as a decrease in platelet activity or a reduction in cholesterol level. However, the inflammatory theory of atherosclerosis resulted in the examination of these therapies' potential anti-inflammatory effects as the usefulness and effectiveness of the drugs were assessed. In their paper “Simvastatin Effects on Inflammation and Platelet Activation Markers in Hypercholesterolemia”, C. Barale et al. confirm that, in addition to its lipid-lowering effect, simvastatin acts as an anti-inflammatory and platelet-inhibiting agent. In their study, the simvastatin also improved the parameters of ED in patients with hypercholesterolemia. These changes were most likely a part of the pleiotropic effect attributed to the 3-hydroxy-3-methylglutaryl coenzyme A (HMG-CoA) reductase inhibitors. The authors also suggest that a high LDL cholesterol level, which can be controlled by statins, could be responsible for platelet hyperactivity.

In their paper, P. A. Maranhão et al. demonstrate that ingestion of a high-fat meal involves the impairment of microvascular function in both obese and normal-weight women. High-fat meal intake can acutely impair microvascular function by inducing hyperinsulinemia, endothelial activation, and inflammation. This mechanism worsened ED in the obese women and induced the impairment of endothelial function in the normal-weight women. Due to the key role of ED in atherosclerosis, these findings may also explain the association between ingestion of a high-fat meal and atherosclerosis.

In their review paper, J. Gawrys et al. present the interactions between the cyclooxygenase metabolic pathway and the renin-angiotensin-aldosterone systems (RAAS) in the context of endothelial function balance disturbance. Based on available data, their brief review also summarises the data regarding usefulness and safety of the Acetylsalicylic acid (ASA) combination with drugs that act directly on RAAS.

In another review paper, M. Jakubowski et al. outline the current knowledge regarding the role of human platelet carbonic anhydrase II (CAII) in regulating platelet function. The paper also describes the consideration of this enzyme as a potential drug target and important pathophysiological chain in platelet-related disorders.

The known result of hyperglycaemia is tissue damage as a consequence of many mechanisms according to the polyol pathway, production of advanced glycation end products (AGEs), protein kinase C, and the hexosamine pathway, which are responsible for overproduction of the reactive oxygen species. Moreover, hyperglycaemia appears to be the major determinant of the diabetic microvascular complications' retinopathy, neuropathy, and nephropathy. Most significantly, kidney disease in patients with diabetes aggravates hypertension, which can further damage the kidneys and leads to end-stage renal disease. Because there is still no effective treatment for this complication, early diagnosis with treatment plays a crucial role in patient care. Furthermore, factors or molecules that can be detected in the early stages of nephropathy could lead to new opportunities for further treatment and may well also serve as a marker of endothelial damage, with all of its cardiovascular consequences. Such a factor appears to be the novel proangiogenic factor leucine-rich-*α*2-glycoprotein-1 (LRG1), which promotes abnormal angiogenesis within the renal glomerulus.

LRG1-induced abnormal angiogenesis resulting in glomerular fibrosis was observed in mice in a study by S. Haku et al. and is presented in the paper “Early Enhanced Leucine-Rich *α*-2-Glycoprotein-1 Expression in Glomerular Endothelial Cells of Type 2 Diabetic Nephropathy Model Mice”. Because the glomerular LRG1 expression was increased before the vascular endothelial growth factor (VEGF) and vascular endothelial growth factor receptor-2 (VEGFR-2) increased, the authors suggest that glomerular LRG1 is an earlier marker of abnormal angiogenesis within the renal glomerulus than the mentioned classical growth factors.

For their part, M. A. Ortega et al. examined the behaviour of smooth muscle cells (SMCs) under hypoxic conditions: possible implications on the varicose vein endothelium. The authors share that the muscle cells of people with varicose veins showed levels of the studied markers (hypoxia-inducible factors 1*α* (HIF-1*α*), VEGF, transforming growth factor-*β*1 (TGF-*β*1), and endothelial nitric oxide synthase (eNOS) similar to normal cells subjected to hypoxia). They also demonstrate that, were the hypoxia to continue over the longer term, these cells would no longer have the capacity to react, and the factor that attempts to compensate for the hypoxia (EGLN3) would fail. Additionally, the authors presented totally different status for healthy subjects (controls) compared to the patients with venous insufficiency according to the VEGF level change. Apart from the difference in baseline VFGF concentration in normoxic conditions (higher level in patients with venous insufficiency), in the control group, the expression of VEGF raised in response to long- term hypoxia while in patients with varicose veins this factor's level decreased significantly.

The role of bone marrow-derived endothelial progenitor cells (EPCs) in monocrotaline-induced pulmonary arterial hypertension (PAH) is demonstrated in an original paper by R. Miao et al. In their study, it appeared that an increase in cytosolic free Ca^2+^ is identified as a trigger for promoting both proliferation and vasoconstriction of the pulmonary arterial smooth muscle cells. The balance of cytosolic calcium is controlled by the store-operated Ca^2+^ channels (SOC) in human EPCs, which is mediated by the Orai and the canonical transient receptor potential channels. The results of the study suggest that the association between the low expression of the major mediators of SOC and Ca^2+^ homeostasis may lead to PAH.

Endothelial function in children with acute lymphoblastic leukaemia (ALL) may predict he clinical outcome. A. Doroszko et al. demonstrate that high baseline vascular endothelial growth factor (VEGF) and soluble E-selectin levels, along with a significant increase in plasminogen activator inhibitor-1 (PAI-1) and low initial soluble intercellular adhesion molecule 1(sICAM-1) levels, are predictive of poor prognosis in ALL children.

The editors of the special issue, which is dedicated to the new insights to the endothelial function and its pathological states, strongly believe that the knowledge contained in this issue will mobilize many scientists to the further work as well as contributing to a better understanding human's physiology and pathology.

